# Publisher Correction: A meiosis-specific BRCA2 binding protein recruits recombinases to DNA double-strand breaks to ensure homologous recombination

**DOI:** 10.1038/s41467-019-08995-4

**Published:** 2019-02-25

**Authors:** Jingjing Zhang, Yasuhiro Fujiwara, Shohei Yamamoto, Hiroki Shibuya

**Affiliations:** 1 Department of Chemistry and Molecular Biology, University of Gothenburg, SE-40530 Gothenburg, Sweden; 2 Institute for Quantitative Biosciences, University of Tokyo, 1-1-1 Yayoi, Tokyo, 113-0032 Japan; 3 Graduate Program in Bioscience, Graduate School of Science, University of Tokyo, Hongo, Tokyo 113-0033 Japan

Correction to: *Nature Communications;* 10.1038/s41467-019-08676-2; published online: 13 February 2019

The original version of this Article contained errors in Fig. 5. In panel g, the male and female symbols preceding each genotype were inadvertently converted to ‘B‘ and ‘≅‘, respectively. These errors have been corrected in both the PDF and HTML versions of the Article.

