# Supplementary material for: A meiosis-specific BRCA2 binding protein recruits recombinases to DNA double-strand breaks to ensure homologous recombination
Source: Nat Commun. 2019 Feb 13;10:722. doi: 10.1038/s41467-019-08676-2 (PMC6374363; doi:10.1038/s41467-019-08676-2)
Supplement: Supplementary file 1 — Supplementary Information [file 41467_2019_8676_MOESM1_ESM.pdf]

Supplementary information

A meiosis-specific BRCA2 binding protein recruits recombinases to DNA double-strand breaks to ensure homologous recombination

Zhang. J, Fujiwara. Y et al.

```
M.musculus         1 MAATVGDGSGTEEACRNMESKEEFVKVRKKDLERLTTEVMQIRDFLPRILNGELLESFQK
R.norvegicus       1 MAAIVGERSGTEEACRHMESKEEFVKVRKKDLERLTTEVMQIRDFLPRILNGELLESFQK
H.sapiens          1 ----MGEAGAAEEACRHMGTKEEFVKVRKKDLERLTTEVMQIRDFLPRILNGEVLESFQK
P.troglodytes      1 ----MGEAGAAEEACRHMGTKEEFVKVRKKDLERLTTEVMQIRDFLPRILNGEVLESFQK
C.lupus            1 ----MGETGGAEEACGHVGTKEEFVKVRKKDLERLTTEVMQIRDFLPRILNGEVLESFQK
B.taurus           1 ----MNEAGGAEETCRHMGTKEEFVKVRKKDLERLTTEVMQIRDFLPRILNGEVLETFQK
X.tropicalis       1 ---------------MAGSSGAEFIRIRRRDLERLTTEVMQMKDFLPKILNPELVEIVQR
D.rerio            1 -MKAVDPVNVPSSSAVQVVGSDGFVQVRKRDLERLEAEVRTLRELMPKVINSDLIDTIHE

M.musculus        61 LKMVEKNLERKEQELEQLIMDREHFKARLETAQADSGREKKEKLALRQQLNEAKQQLLQQ
R.norvegicus      61 LKIVEKNLERKEQELEQLRLDCEHFKARLETALADSGREKKEKLALRQQLNEAKQQLLQQ
H.sapiens         57 LKIVEKNLERKEQELEQLKMDCEHFKARLETVQADNIREKKEKLALRQQLNEAKQQLLQQ
P.troglodytes     57 LKIVEKNLERKEQELEQLKMDCEHFKARLETVQADDIREKKEKLALRQQLNEAKQQLLQQ
C.lupus           57 LKIVEKNLERKEQELEQLRMDCEHFKARLETVQADSMRDKKEKLALRQQLNEAKQQLLQQ
B.taurus          57 LKIVEKNLERKEQELEQLRMDCEHFRARLESVQADSVREKKEKLALRQQLNEAKQQLLQQ
X.tropicalis      46 LEQAETALEKKT-------LDCDHLMARLEAAQSECVRERQEKLSLVSQVSSLREQSVQQ
D.rerio           60 GRSLDAFQELCDRE-QQERENCLHIQTRLDAALNECQNEKQEKLVLKQQLWECRGQLQQQ

M.musculus       121 AEYCTQMGAVTCTLLWGVSSSEEVVKTILGGDKALKFFNITGQTMESFVKSLDG---DVK
R.norvegicus     121 AEYCTQMGAVTCTLLWGVSSSEEVVKAILGGDKALKFFNITGQTMESFVKSLDG---DVK
H.sapiens        117 AEYCTEMGAAACTLLWGVSSSEEVVKAILGGDKALKFFSITGQTMESFVKSLDG---DVQ
P.troglodytes    117 AEYCTEMGAAACTLLWGVSSSEEVVKAILGGDKALKFFSITGQTMESFVKSLDG---DVQ
C.lupus          117 AEYCTEMGAAACTLLWGVSSSEDVVKAILGGDKALKFFSITGQTMESFVKSLDG---DVK
B.taurus         117 AEYCTELGAAACTILWGVSSSEEVVKAILGGDKALKFFNITGQTMESFVKSLDG---DVK
X.tropicalis      99 AEYCSHMGAAACTLLWGVSHKEEVVLSILGGSKAPMFFSLAAQTLSSFVRSLTSE--QPQ
D.rerio          119 KDFCTELGASCCTLLWSASQKEEAIRDILANGKLEDFLSIAGQTLETFIKLLVDEAKPQQ

M.musculus       178 EVDSDENQFVFALAGIVTNVAAIACGREFLVNSSRVLLDTMLQLLGDLKPGQCTKLKVLM
R.norvegicus     178 EVDSDENQFVFALAGIVTNVAAVACGREFLVNSSQVLLDTMLQLLGDLKPGQCTKLKVLM
H.sapiens        174 ELDSDESQFVFALAGIVTNVAAIACGREFLVNSSRVLLDTILQLLGDLKPGQCTKLKVLM
P.troglodytes    174 ELDSDESQFVFALAGIVTNVAAIACGREFLVNSSRVLLDTILQLLGDLKPGQCTKLKVLM
C.lupus          174 ELDSDENQFVFALAGIVTNVAAIACGREFLVNSSRVLLDTILQLLGDLKPGQCTKLKVLL
B.taurus         174 ELDSDENQFVFALAGIVTNVAAIASGREFLVNSSRVLLDTILQLLGDLKPGQCTRLKVLM
X.tropicalis     157 DEENEESHFVLGLAGTVTNVAAVSSGREFLVSRCQDLLETWIQLLEEIGLGSCCRLRVLL
D.rerio          179 SDNSHEHNFVLALAGVVTNIAAVSCGRDFLSSSAHILLGTMLQLLGLMKPGVFPKLKVLM

M.musculus       238 LMSLYNVSINSKGLKYITESPGFIPLLWWLLSDPDAEVCLHTLRLIQSVVLEPDVFSKVA
R.norvegicus     238 LMSLYNVSINSKGLKYISESPGFIPLLWWLLSDPDAEVCLHAVRLIQSVVLEPDVFSKVA
H.sapiens        234 LMSLYNVSINLKGLKYISESPGFIPLLWWLLSDPDAEVCLHVLRLVQSVVLEPEVFSKSA
P.troglodytes    234 LMSLYNVSINLKGLKYISESPGFIPLLWWLLSDPDAEVCLHVLRLVQSVVLEPEVFSKSA
C.lupus          234 LMSLYNVSINLKGLKYISESPGFIPLLWWLLSDPDAEVCLHALRLVQSLVLEPEVLSKSA
B.taurus         234 LMSLYNVSINLKGLKYISESPGFIPLLWWLLSDPDAEVCLHVLRLVQSVVLEPEVFSRSA
X.tropicalis     217 LMSLYNVSINQKGLSWMSNNHKLISQVQRLLTDPDPEVCLHALRLFQSVVLEPEVLPRLK
D.rerio          239 LMALYNTTINVNGLKFISERPELLPLMCHLLEDPDPEVCLQSLRLLQSLILEREIMPGMA

M.musculus       298 SELQSSLPLQRILAMSKSRNSHLQSAAQELLEDLRALDCNV-------------------
R.norvegicus     298 SELQSSLPLQRILAMSKSRNSHLQSAARELLEDLRALDCSV-------------------
H.sapiens        294 SEFRSSLPLQRILAMSKSRNPRLQTAAQELLEDLRTLEHNV-------------------
P.troglodytes    294 SEFRSSLPLQRILAMSKSRNPRLQTAAQELLEDLRTLEHHV-------------------
C.lupus          294 SEFRSSLPLQRILAMAKSRNHHLQTVAQELLDDLRALERDV-------------------
B.taurus         294 SEFRSSLPLQRILAMAKSRNPHLQTVAQELLEDLRALERDV-------------------
X.tropicalis     277 ADLQESVG--HIVALSQSRNRQLQSEACELLEEVKVLQADA-------------------
D.rerio          299 TDFRRSFPLSRINHLVSSCHPTLKQTAQETLEDLAAVSKLCSGGTMTSKVELQPQLDNNS

M.musculus           -------
R.norvegicus         -------
H.sapiens            -------
P.troglodytes        -------
C.lupus              -------
B.taurus             -------
X.tropicalis         -------
D.rerio          359 NTRNILY
```

Supplementary Fig. 1. Sequence alignment of MEILB2 homologs in vertebrates.
Sequence data are from the NCBI protein database.

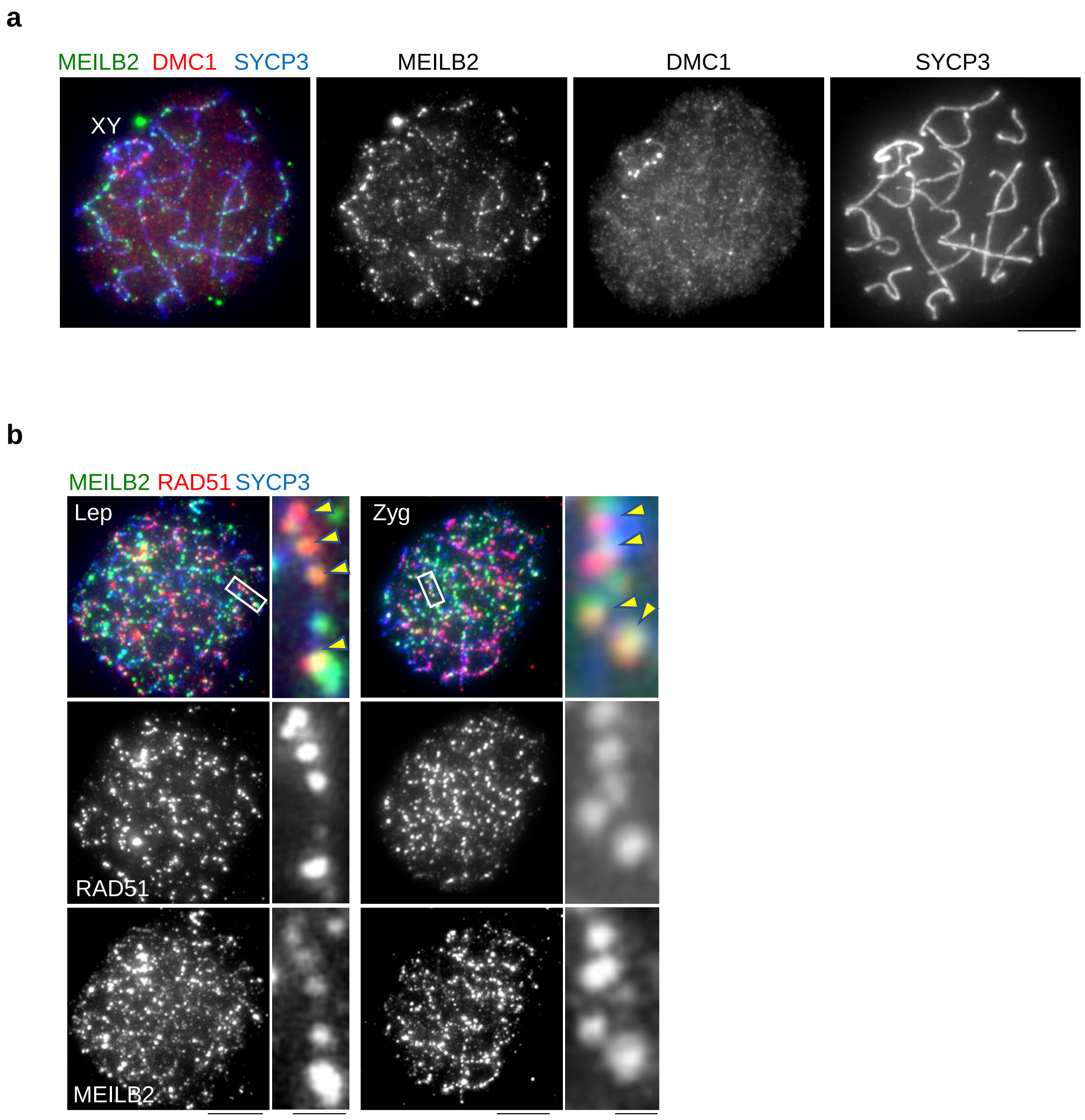

Supplementary Fig. 2. Characterization of MEILB2 localization in spermatocytes.
(**a**) Early pachytene spermatocytes from WT stained with the indicated antibodies and DAPI. The XY chromosomes are highlighted. (**b**) WT spermatocytes stained with the indicated antibodies and DAPI. The colocalizing foci along the chromosome axis are highlighted by yellow arrowheads. The quantification of co-localization was performed using four late leptotene cells and eight zygotene cells pooled from two mice. The axis-associated foci were counted. Scale bars, 5 µm and 1 µm (magnified panel). Source data are provided as a Source Data file.

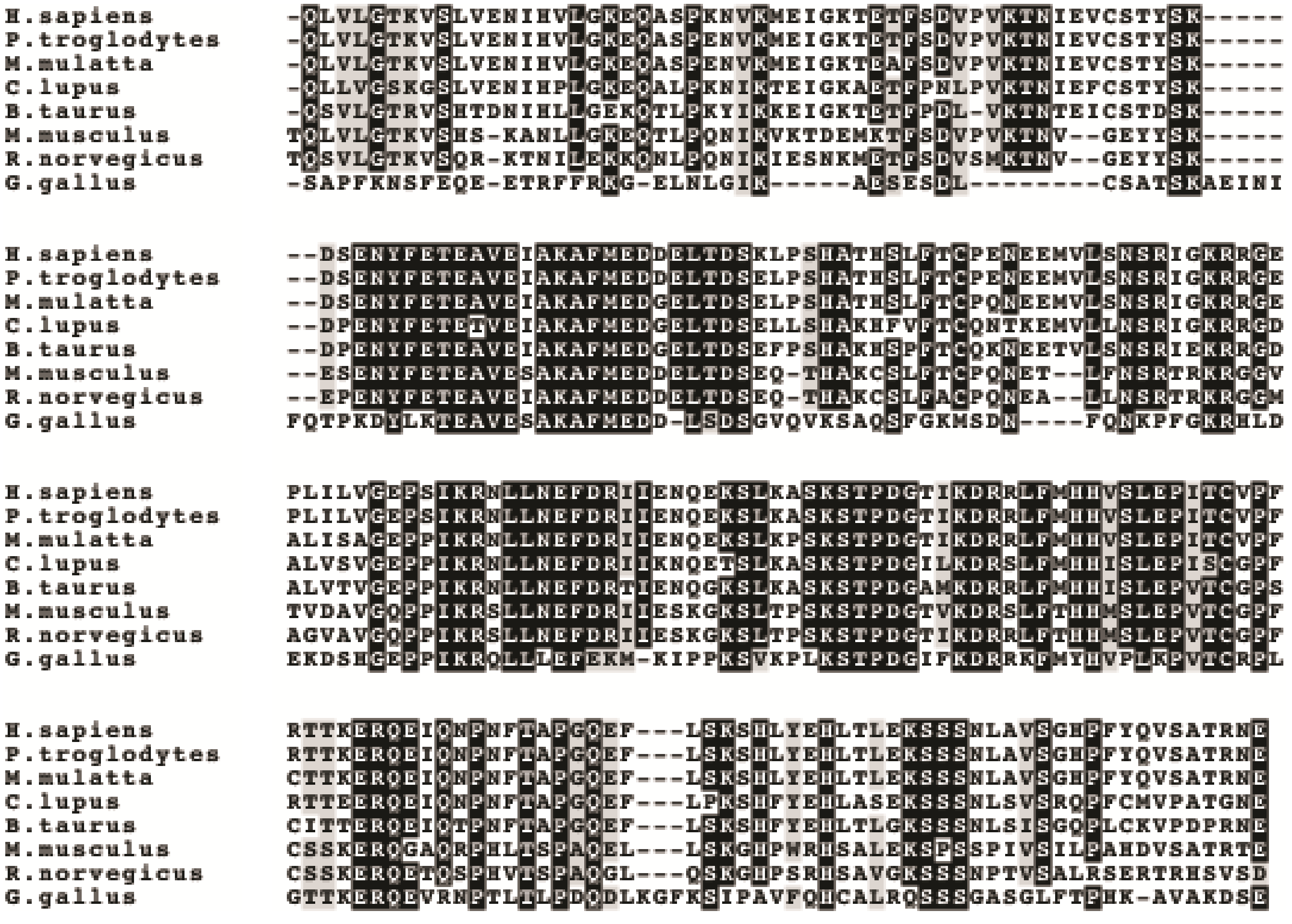

Supplementary Fig. 3. Sequence alignment of BRCA2 MBD in vertebrates.
Sequence data are from the NCBI protein database. *H. sapiens* (NP_000050.2, 2164-2391 a.a.), *P. troglodytes* (XP_509619.2, 2164-2391 a.a.), *M. mulatta* (XP_001118184.2, 2106-2333 a.a.), *C. lupus* (NP_001006654.2, 2181-2408 a.a.), *B. taurus* (XP_002691853.1, 2171-2397 a.a.), *M. musculus* (NP_033895.2, 2117-2339 a.a.), *R. norvegicus* (NP_113730.2, 2132-2354 a.a.), *G. gallus* (NP_989607.2, 2124-2339 a.a.)

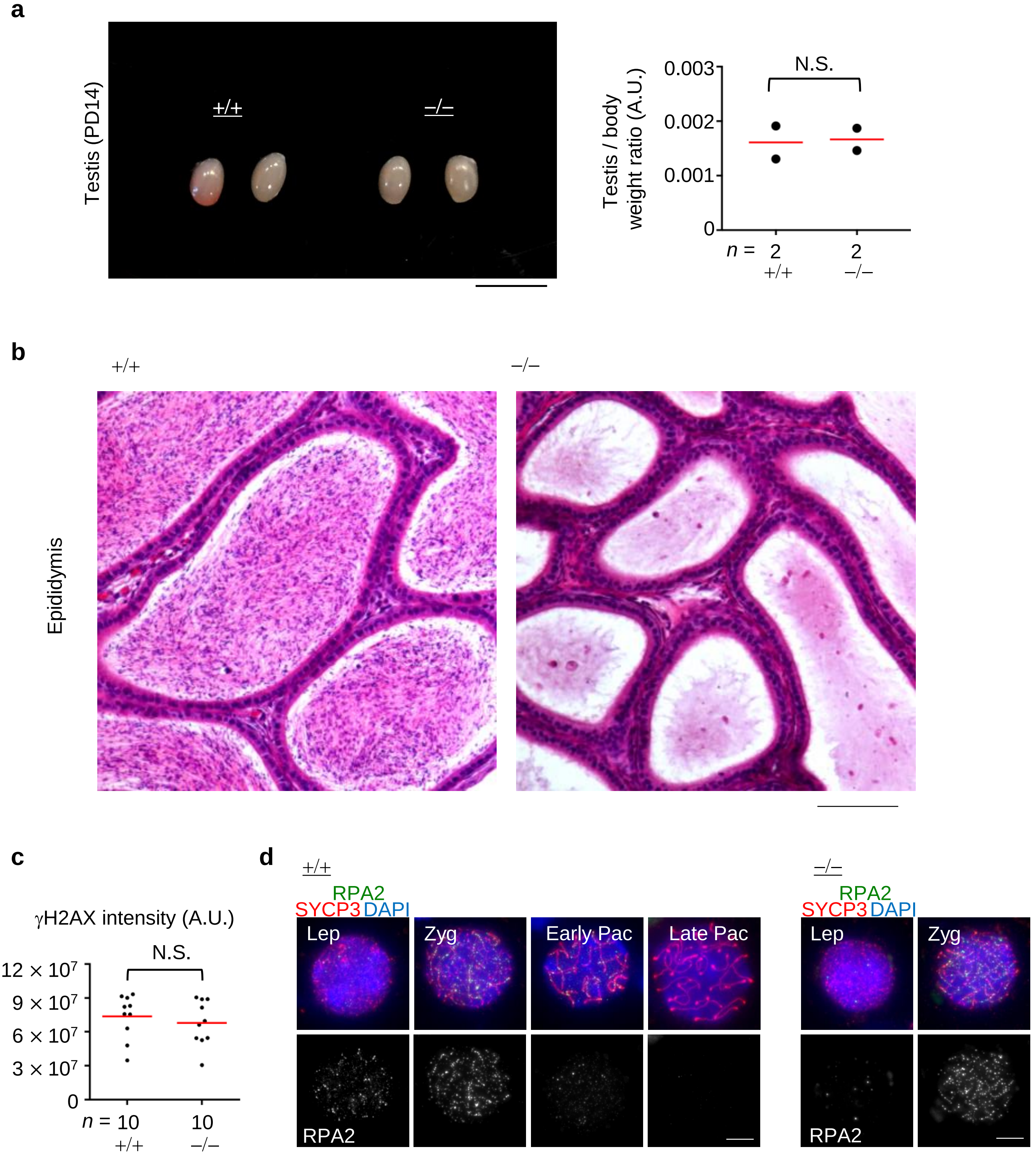

Supplementary Fig. 4. Characterization of *Meilb2*–/– male phenotype.
(**a**) Juvenile testes from WT (+/+) and *Meilb2* KO (–/–) males at PD14. The graph shows the quantification of testis / body weight ratio. *n* shows analyzed mouse number. The mean value is shown as a red bar. Scale bar, 2 mm. (**b**) Epididymis sections from 8-week-old WT (+/+) and *Meilb2*KO (–/–) male mice stained with hematoxylin and eosin. Scale bar, 100 μm. (**c**) The signal intensity of γH2AX intensity in zygotene spermatocytes from WT (+/+) and *Meilb2* KO (–/–) males. The mean value is shown as a red bar. *n* shows the analyzed zygotene spermatocytes number pooled from three mice for each genotype. WT (+/+) and *Meilb2* KO (–/–) samples were prepared and stained at the same time on the same glass slide. (**d**) Spermatocytes from WT (+/+) and *Meilb2* KO (–/–) males stained with the indicated antibodies and DAPI. Each meiotic prophase I substage is shown. Lep; leptotene, Zyg; zygotene, Pac; pachytene, Dip; diplotene. Analyses were with two-tailed *t*-tests. N.S., not significant. Scale bars, 5 μm. Source data are provided as a Source Data file.

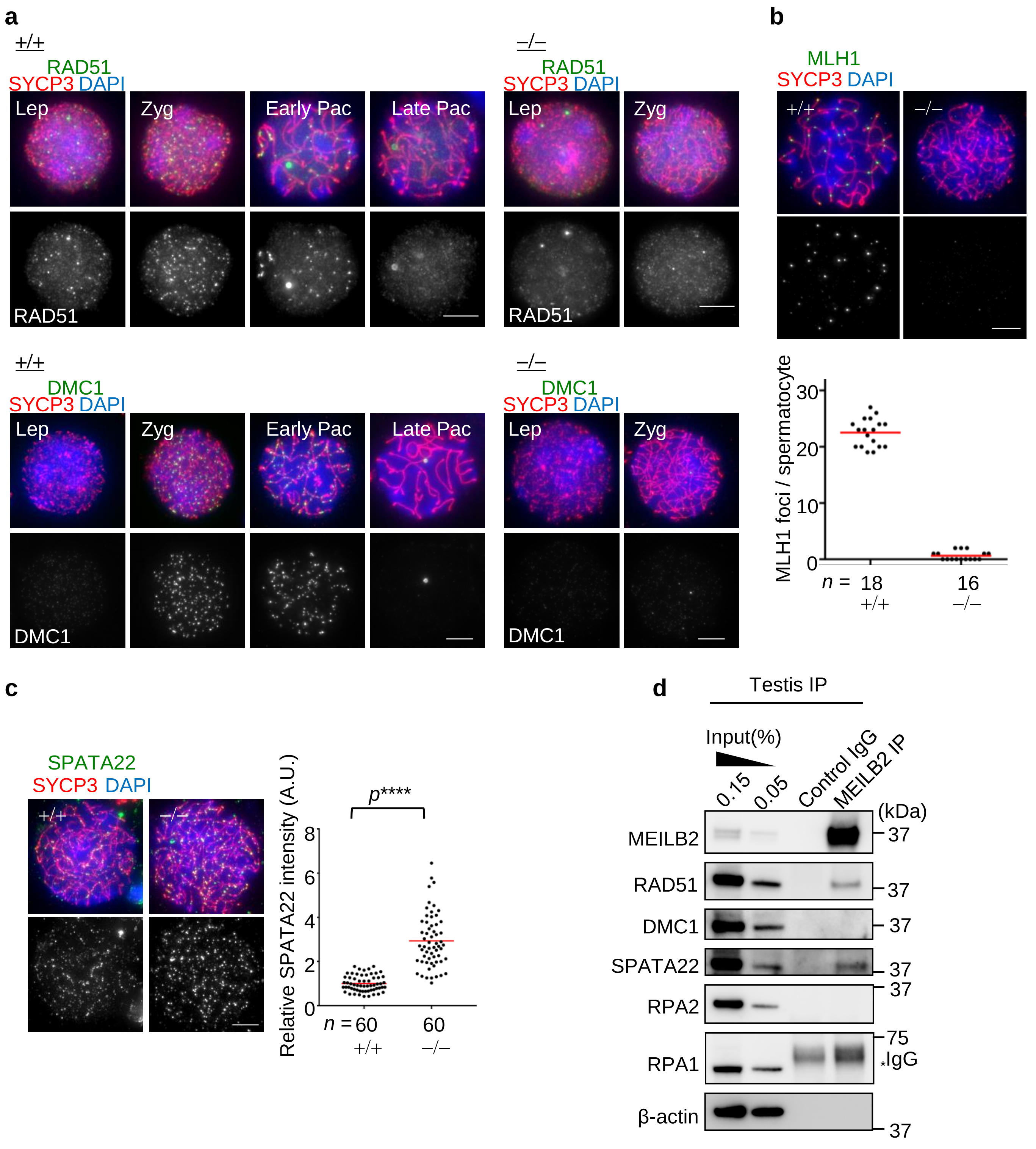

Supplementary Fig. 5. RAD51 and DMC1 foci disappeared in *Meilb2* ^Δ^ spermatocytes resulting in the complete loss of a destined crossover marker.

(**a**) Spermatocytes from WT (+/+) and *Meilb2* KO (–/–) males stained with the indicated antibodies and DAPI. Each meiotic prophase I substage is shown. Lep; leptotene, Zyg; zygotene, Pac; pachytene, Dip; diplotene. (**b**) Pachytene spermatocytes from WT (+/+) and zygotene-arrested spermatocytes from *Meilb2* KO (–/–) males stained with the indicated antibodies and DAPI. The graph shows the number of MLH1 foci associated with the chromosome axes. The mean value is shown as a red bar. *n* shows the analyzed spermatocyte number pooled from two mice for each genotype. (**c**) Zygotene spermatocytes from WT (+/+) and *Meilb2* KO (–/–) males stained with the indicated antibodies and DAPI. The graph shows the relative signal intensity of SPATA22 foci normalized to the average values of WT. The mean value is shown as a red bar. *n* shows the analyzed foci number. Twenty randomly selected axis-associated foci were counted from each cell (three cells in total from a single mouse for each genotype). WT (+/+) and *Meilb2* KO (–/–) samples were prepared and stained at the same time on the same glass slide. (**d**) Immunoprecipitates from mouse testis extracts with the MEILB2 antibody or with IgG as the negative control and immunoblotted with the indicated antibodies. The blots with β-actin served as the loading control. * shows IgG bands (heterodimer of heavy and light chains at 75 kDa). All analyses were with two-tailed t-tests. ****$p < 0.0001$. Scale bars, 5 μm. Source data are provided as a Source Data file.

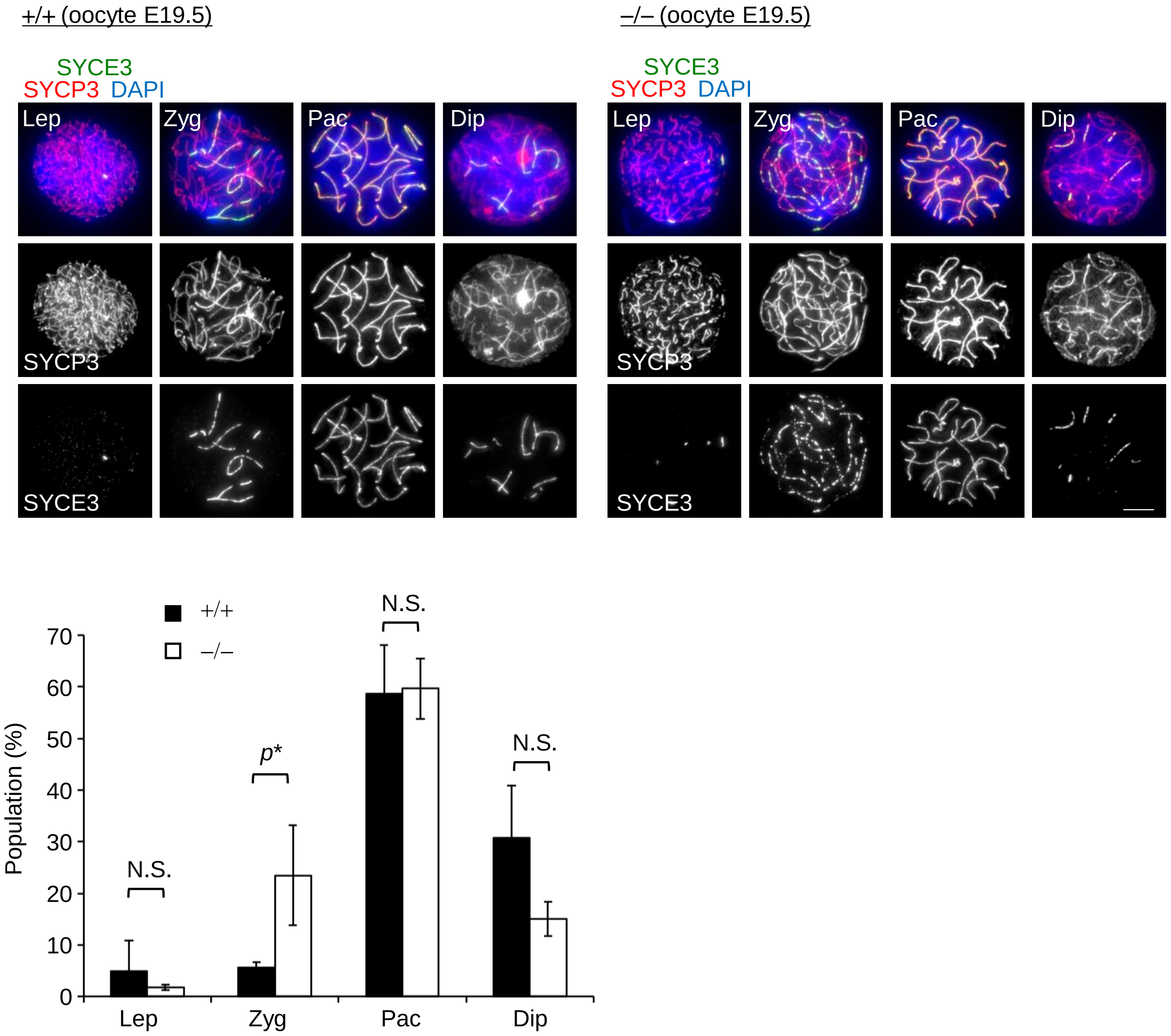

Supplementary Fig. 6. Oocytes from Meilb2 $^{\Delta}$ ovaries contained all meiotic prophase substages with a slight zygotene accumulation.

Oocytes from WT (+/+) and *Meilb2* KO (–/–) females at E19.5 stained with the indicated antibodies and DAPI. Each meiotic prophase I substage is shown. SYCP3-positive oocytes (194 cells for +/+ and 468 cells for –/–) were classified into the following substages: Lep, leptotene (no SYCE3); Zyg, zygotene (partially assembled SYCE); Pac, pachytene (fully assembled SYCE) and Dip, diplotene (disassembled SYCE). The mean values of four independent experiments for (+/+) (from four embryos from four individual mice) and two independent experiments for (–/–) (from two embryos from two individual mice) are shown. Error bars show the SD. All analyses were with two-tailed t-tests. N.S., not significant. *$p < 0.05$. Scale bar, 5 µm. Source data are provided as a Source Data file.

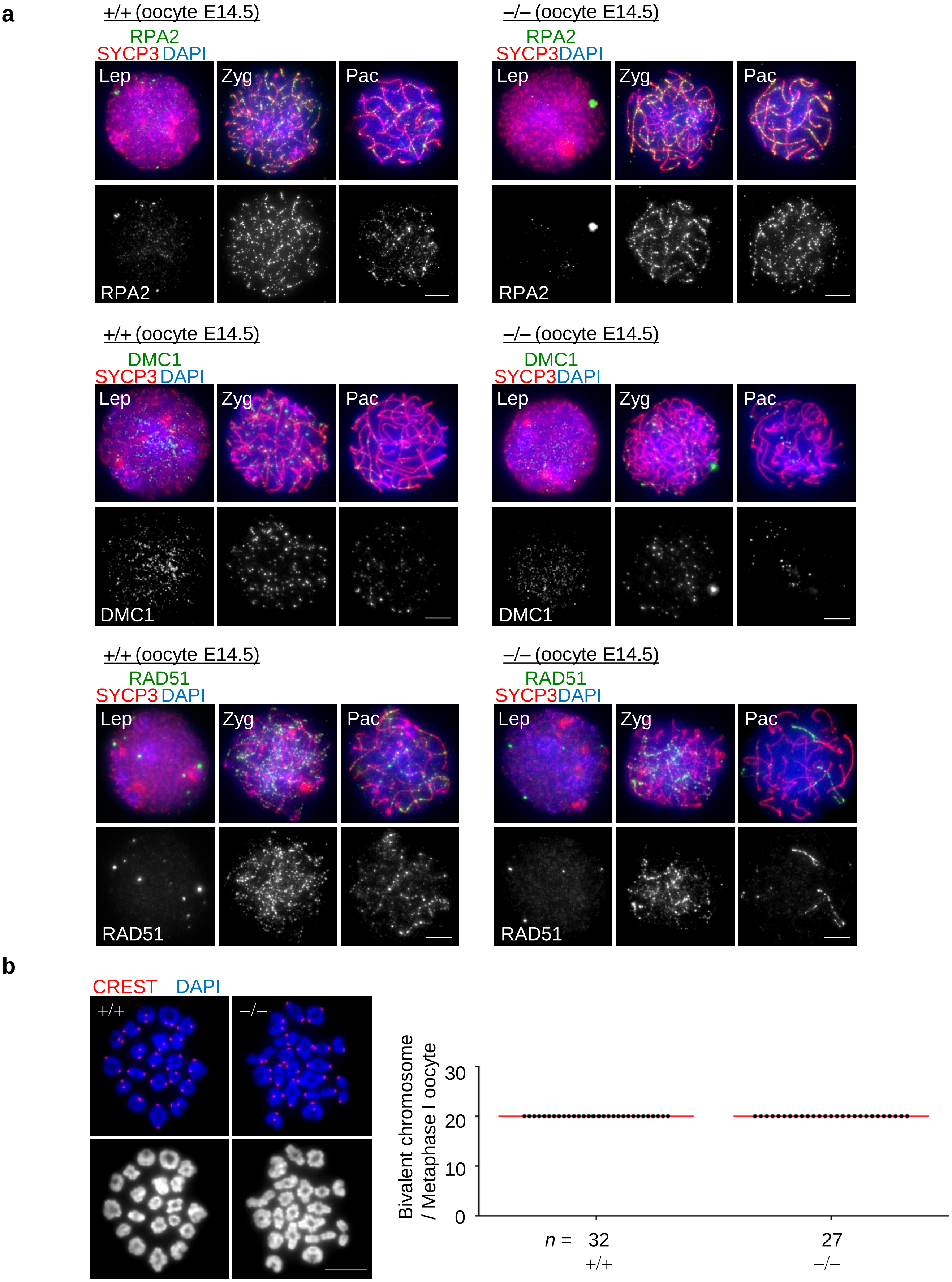

Supplementary Fig. 7. Characterization of *Meilb2* $^{\Delta}$ female phenotype.
(**a**) Oocytes from WT (+/+) and *Meilb2* KO (–/–) females at E14.5 stained with the indicated antibodies and DAPI. Each meiotic prophase I substage is shown. (**b**) Metaphase I oocyte from WT (+/+) and *Meilb2* KO (–/–) females at PD25 stained with the indicated antibodies and DAPI. The numbers of bivalent chromosomes were quantified. The mean value is shown as a red bar. *n* shows the analyzed oocyte number pooled from three mice for each genotype. Scale bars, 5 µm. Source data are provided as a Source Data file.

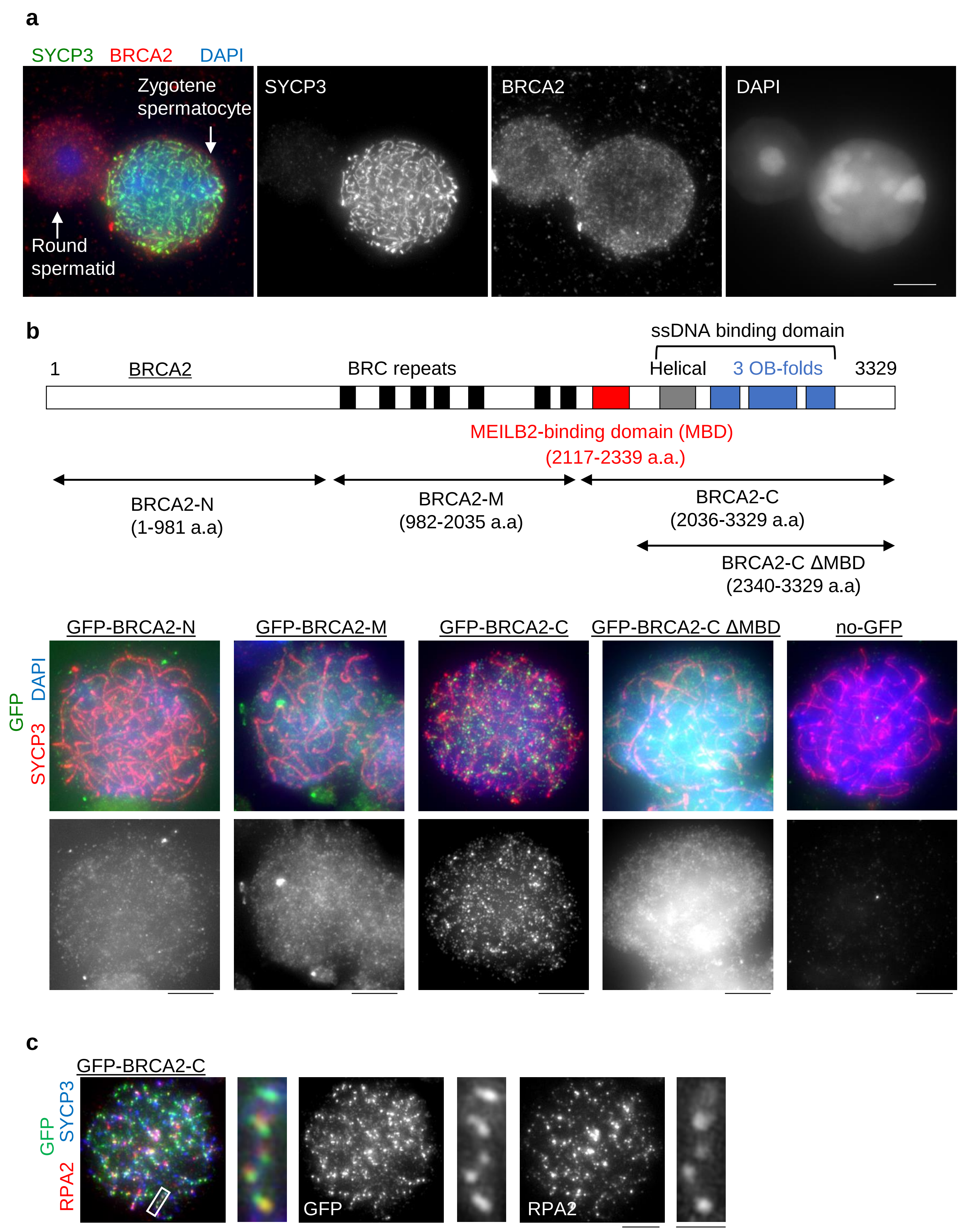

Supplementary Fig. 8. Characterization of BRCA2 localization in spermatocytes.
(**a**) WT testis cell suspension stained with the indicated antibodies and DAPI. A zygotene spermatocyte and round spermatid are indicated. Cloudy BRCA2 staining in the spermatocyte was hardly distinguishable from the background signal seen in the round spermatid. (**b**) The domain conformation of BRCA2. The bottom pictures show WT zygotene spermatocytes expressing GFP-BRCA2 truncations stained with the indicated antibodies and DAPI. (**c**) WT zygotene spermatocytes expressing GFP-BRCA2-C stained with the indicated antibodies and DAPI. The quantification of co-localization was performed using nine zygotene cells pooled from three electroporated mice. The axis-associated foci were counted. Scale bars, 5 µm and 1 µm (magnified panel). Source data are provided as a Source Data file.

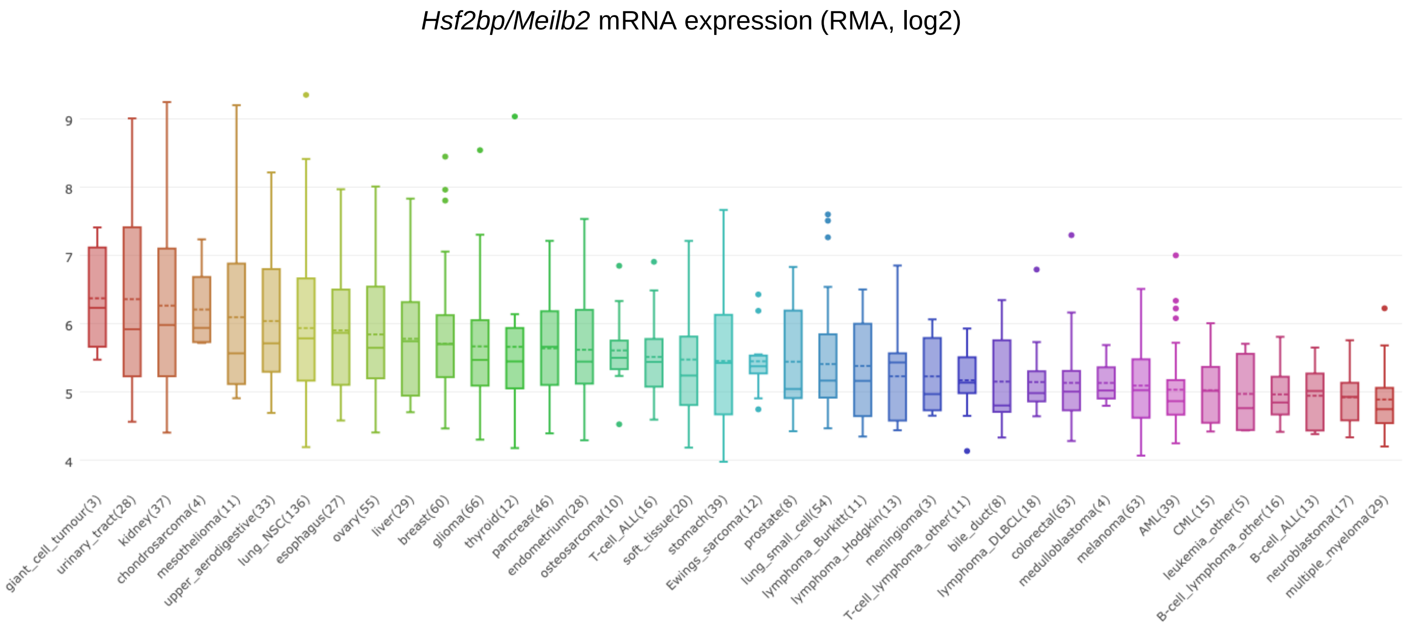

Supplementary Fig. 9. Expression of *Hsf2bp/Meilb2* gene in human cancer cell lines from CCLE database.
The boxplot is sorted and colored by average distribution of a gene's expression in a lineage. The number next to the lineage name indicates how many cell lines are in the lineage. The highest average distribution is on the left and is colored in red. The dashed line within a box is the mean.
