# Supplementary material for: A meiosis-specific BRCA2 binding protein recruits recombinases to DNA double-strand breaks to ensure homologous recombination
Source: Nat Commun. 2019 Feb 13;10:722. doi: 10.1038/s41467-019-08676-2 (PMC6374363; doi:10.1038/s41467-019-08676-2)
Supplement: Supplementary file 3 — Reporting Summary [file 41467_2019_8676_MOESM3_ESM.pdf]

nature research

Corresponding author(s): Hiroki Shibuya

Last updated by author(s): 20019-01-11

Reporting Summary

Nature Research wishes to improve the reproducibility of the work that we publish. This form provides structure for consistency and transparency in reporting. For further information on Nature Research policies, see Authors & Referees and the Editorial Policy Checklist.

Statistics

For all statistical analyses, confirm that the following items are present in the figure legend, table legend, main text, or Methods section.

n/a	Confirmed	
☐	☒	The exact sample size (*n*) for each experimental group/condition, given as a discrete number and unit of measurement
☐	☒	A statement on whether measurements were taken from distinct samples or whether the same sample was measured repeatedly
☐	☒	The statistical test(s) used AND whether they are one- or two-sided<br>*Only common tests should be described solely by name; describe more complex techniques in the Methods section.*
☐	☒	A description of all covariates tested
☐	☒	A description of any assumptions or corrections, such as tests of normality and adjustment for multiple comparisons
☐	☒	A full description of the statistical parameters including central tendency (e.g. means) or other basic estimates (e.g. regression coefficient) AND variation (e.g. standard deviation) or associated estimates of uncertainty (e.g. confidence intervals)
☐	☒	For null hypothesis testing, the test statistic (e.g. *F*, *t*, *r*) with confidence intervals, effect sizes, degrees of freedom and *P* value noted<br>*Give P values as exact values whenever suitable.*
☒	☐	For Bayesian analysis, information on the choice of priors and Markov chain Monte Carlo settings
☒	☐	For hierarchical and complex designs, identification of the appropriate level for tests and full reporting of outcomes
☒	☐	Estimates of effect sizes (e.g. Cohen's *d*, Pearson's *r*), indicating how they were calculated

Our web collection on statistics for biologists contains articles on many of the points above.

Software and code

Policy information about availability of computer code

Data collection	N/A
Data analysis	Excel (16.16.5), GraphPad Prism (7.0c), SoftWoRx (5.5.5)

For manuscripts utilizing custom algorithms or software that are central to the research but not yet described in published literature, software must be made available to editors/reviewers. We strongly encourage code deposition in a community repository (e.g. GitHub). See the Nature Research guidelines for submitting code & software for further information.

Data

Policy information about availability of data

All manuscripts must include a data availability statement. This statement should provide the following information, where applicable:

- Accession codes, unique identifiers, or web links for publicly available datasets
- A list of figures that have associated raw data
- A description of any restrictions on data availability

The data supporting the findings of this study are available from the corresponding author upon reasonable request. The source data underlying Figs 1d-f, 3c, e, g, h, i, 4c, d, e, g, h, 5a, c, d, e, f, g, 6c-e and Supplementary Figs 2b, 4a, c, 5b, c, 6, 7b, and 8c are provided as a Source Data file.

Field-specific reporting

Please select the one below that is the best fit for your research. If you are not sure, read the appropriate sections before making your selection.

☒ Life sciences ☐ Behavioural & social sciences ☐ Ecological, evolutionary & environmental sciences

Life sciences study design

All studies must disclose on these points even when the disclosure is negative.

Sample size	For all experiments, sample size pre-calculation was not performed. In general, experiments were performed 3 times (n=3), otherwise indicated. These numbers are sufficient to obtain statistical differences or effect sizes.
Data exclusions	No data was excluded.
Replication	All experiments were reliably reproduced as stated in the text.
Randomization	We used genetically engineered mice, and each group were allocated based on the genotypes.
Blinding	Investigators were not blinded as this is not applicable to our study.

Reporting for specific materials, systems and methods

We require information from authors about some types of materials, experimental systems and methods used in many studies. Here, indicate whether each material, system or method listed is relevant to your study. If you are not sure if a list item applies to your research, read the appropriate section before selecting a response.

Materials & experimental systems

n/a	Involved in the study	
☐	☒	Antibodies
☐	☒	Eukaryotic cell lines
☒	☐	Palaeontology
☐	☒	Animals and other organisms
☒	☐	Human research participants
☒	☐	Clinical data

Methods

n/a	Involved in the study	
☒	☐	ChIP-seq
☒	☐	Flow cytometry
☒	☐	MRI-based neuroimaging

Antibodies

Antibodies used	The following antibodies were used: rabbit antibodies against MEILB2 (this study), GFP (Invitrogen; A11122), γH2AX (Abcam; ab11174), DMC1 (Santa Cruz Biotechnology; sc-22768), RAD51 (Thermo Fisher Scientific; PA5-27195), SPATA22 (Proteintech Group Inc; 16989-1-AP), and SYCE3; mouse antibodies against MEILB2 (this study), DMC1 (this study), β-actin (Sigma; A2228-100UL), MLH1 (BD Biosciences; 51-1327GR), and MYC (MBL; M192-3); rat antibody against RPA2 (Cell Signaling Technology; 2208); sheep antibody against BRCA2; chicken antibody against SYCP3 (this study); guinea pig antibody against histone H1T; and human anti-centromere antibody (Antibodies Incorporated; 15-234-0001).
Validation	Each experiment had appropriate controls to validate the antibodies. Commercially available antibodies were validated by the supplier and by us using appropriate controls where needed. Non-commercial antibodies provided by colleagues were validated in their initial studies describing the corresponding antibody.

Eukaryotic cell lines

Policy information about cell lines

Cell line source(s)	B16-F1 (sigma), C2C12 (sigma)
Authentication	These cell lines are purchased from Sigma, and authenticated.
Mycoplasma contamination	We added antibiotic Plasmocin to avoid contamination of mycoplasma and all cells are routinely tested to be mycoplasma free.
Commonly misidentified lines (See ICLAC register)	N/A

Animals and other organisms

Policy information about studies involving animals; ARRIVE guidelines recommended for reporting animal research

Laboratory animals	All WT and KO mice were congenic with the C57BL/6J background. Both male and female was used. The age of the mice is 2 moth old, otherwise indicated in the figure legend.

Wild animals	N/A
Field-collected samples	N/A
Ethics oversight	Animal experiments were approved by the Institutional Animal Care and Use Committee (#1316/18).

Note that full information on the approval of the study protocol must also be provided in the manuscript.
